# Edema Index Predicts Mortality in Patients with Chronic Heart Failure: A Prospective, Observational Study

**DOI:** 10.5334/gh.1287

**Published:** 2024-01-18

**Authors:** Bryan Richard Sasmita, Yuhe Zhao, Ming Gong, Suxin Luo, Bi Huang

**Affiliations:** 1 Department of Cardiology, the First Affiliated Hospital of Chongqing Medical University, 400016, Chongqing, China; 2 Department of Cardiology, Chongqing Seventh People’s Hospital, Chongqing, China

**Keywords:** Chronic Heart Failure, Edema Index, Fluid Congestion, All-cause mortality, Cardiovascular mortality

## Abstract

**Introduction:**

Chronic fluid accumulation or congestion is considered an important pathophysiologic mechanism in heart failure, leading to cardinal symptoms such as dyspnea, pulmonary congestion, and pitting edema. Edema index (EI) recently emerged as a surrogate for extracellular volume status and has been proven to be able to reflect one’s congestion status. In this study, we aimed to evaluate the prognostic value of EI in patients with chronic heart failure (CHF).

**Methods:**

A total of 401 consecutive patients with CHF between August 2019 and October 2021 were prospectively enrolled. EI was obtained by InBody S10. The primary endpoint was long-term all-cause and cardiovascular mortality.

**Results:**

Patients with high EI (>0.397) tended to be older, presented with atrial fibrillation, have higher N-terminal brain natriuretic peptide, and have higher creatinine (all p < 0.05). During a median follow-up of 1200 days, the all-cause and cardiovascular mortality rate was significantly higher in the high EI group compared to the low EI group (all-cause mortality rate 43.8% vs. 30.3%, p < 0.001, and cardiovascular mortality rate 17.5% vs. 13.0%, p < 0.001, respectively). In the multivariate Cox proportional hazard analysis, EI > 0.397 was an independent predictor for both all-cause mortality (HR 1.959; 95% CI 1.304, 2.944; p = 0.001) and cardiovascular mortality (HR 2.051; 95% CI 1.276, 3.296; p = 0.003).

**Conclusions:**

Admission EI could be used as a marker for predicting long-term mortality in patients with CHF, and higher EI was associated with an increased risk of all-cause and cardiovascular mortality. Furthermore, EI-guided management could be a promising therapy in patients with CHF.

## Key Summary Points

### Why carry out the study?

- Chronic fluid accumulation or congestion is considered an important pathophysiologic mechanism in heart failure and has long been associated with a worse prognosis and increased risk of hospitalization.
- Accurate fluid monitoring relies on invasive hemodynamics; however, it is mainly used in critical conditions, while for ordinary patients with heart failure, there is no reliable and objective method for evaluating volume overload.

### What was learned from the study?

- Body impedance analysis can be used as a simple, reliable, and non-invasive tool to evaluate the congestion status of patients with chronic heart failure.
- High admission EI was associated with an increased risk of long-term all-cause and cardiovascular mortality.
- EI-guided management may be a promising way to achieve and maintain euvolemia in patients with chronic heart failure.

## 1. Introduction

Congestive heart failure (HF) is a major cause of cardiovascular mortality and morbidity, characterized by impaired ventricular function and insufficient peripheral blood supply [1]. The reduced blood flow results in the activation of neurohormonal systems, abnormal circulatory hemodynamics, and sodium and water retention [2]. In the early stages, these compensatory mechanisms are beneficial to maintain cardiac output; however, when HF progresses, it becomes detrimental and leads to a vicious cycle, where they become maladaptive, further depress ventricular performance, and increase sodium and water retention [3]. Chronic fluid accumulation or congestion is considered an important pathophysiologic mechanism in HF, leading to cardinal symptoms such as dyspnea, pulmonary congestion, and pitting edema [4]. This volume overload is not only associated with an increased risk of hospitalization but also with a worse prognosis [5, 6]. Therefore, monitoring and proper intervention for volume overload are essential to improve outcomes in patients with HF.

To date, accurate fluid monitoring relies on invasive hemodynamics; however, it is mainly used in critical conditions, while for ordinary patients with HF, there is no reliable and objective method for evaluating volume overload. Bioelectrical impedance analysis (BIA) has recently been suggested as an objective, non-invasive, and rapid method for evaluating body fluid status [7]. It can be accurately performed at the bedside with commercial equipment. The multifrequency analyzer can obtain accurate data on body composition, including intracellular water (ICW), extracellular water (ECW), total body water (TBW), lean body mass (LBM), body fat mass, and other body compartments [7]. The ratio of ECW to TBW, also known as edema index (EI), is recognized as a surrogate for extracellular volume status. Previous studies have shown that EI not only correlates with the severity of acute HF [8], but also serves as a prognostic biomarker in patients with acute HF [8], cancer [9], and peritoneal dialysis [10]. However, whether EI has a similar predictive value in chronic heart failure (CHF) remains unclear. Accordingly, this prospective study was conducted to elucidate the possibility of EI as a new prognostic marker in patients with CHF.

## 2. Methods

### 2.1 Study design

This study was a single-center, prospective observational study designed to evaluate the usefulness of EI for evaluating the prognosis in patients with CHF. This study conformed to the principles outlined in the Declaration of Helsinki and was approved by the institutional ethics review committee of The First Affiliated Hospital of Chongqing Medical University. All patients have provided written informed consent.

### 2.2 Participants

A total of 401 consecutive patients from August 2019 to October 2021 were admitted and enrolled in this study due to CHF. The eligible patients were men and women aged 18 years and older who presented with stable symptomatic CHF of four or more weeks’ duration. The main exclusion criteria were acute HF, congenital heart disease, septic shock, hypovolemic shock, and anaphylactic shock.

### 2.3 Data collection

Baseline characteristics, including age, gender, clinical presentations, comorbidities, and medical histories, were collected. Echocardiography was performed within 24 hours of admission. Blood samples were collected immediately after admission and were tested in the central laboratory. The routine blood test was performed by the Shanghai Sysmex XN-1000 automatic blood cell analyzer. BIA was performed using InBody S10 (InBody China, Beijing, China).

### 2.4 BIA

BIA was performed within eight hours after admission using InBody S10 multi-frequency and segmental analyses. Eight electrodes were attached to the patient’s body—four on the dorsal of the wrists and the bases of the middle fingers, and the other four on the anterior surfaces of the ankles and the bases of the middle toes [11]. Thirty impedance measurements were made by analyzing the conductance of the electrical current across five body segments (legs, arms, and trunk) at multiple frequencies (1, 5, 50, 250, 500, and 1000 kHz). Low-frequencies mainly pass through ECW, enabling measurement of the water content outside of the cells, while high-frequencies penetrate cell membranes and flow along the entire body of water. Therefore, measurement of multiple frequencies allows for an accurate estimation of the water contents inside and outside cells, allowing estimation of the EI.

### 2.5 Outcomes

The primary endpoint of this study was long-term all-cause and cardiovascular mortality.

### 2.6 Statistical analysis

Data were expressed as median and interquartile boundary values or mean ± standard deviation (SD) as appropriate. The comparison of variables in baseline characteristics was calculated with the Pearson X^2^ test, Fisher’s exact test, or Mann-Whitney U test, as appropriate. The area under the curve (AUC) of all-cause and cardiovascular mortality was calculated by the receiver operating characteristic curve (ROC) to determine the predictive value of EI. Patients were then divided into two groups according to the cut-off value of EI determined by Youden’s index. Kaplan-Meier (KM) curves were constructed, followed by the log-rank test.

Logistic and linear regression analyses were used to analyze the association between age with ICW, ECW, TBW, and EI. A Cox regression analysis was performed to analyze the independent association between EI and the prognosis of patients with CHF. The univariate Cox regression model was constructed based on the EI cut-off value, while the multivariate Cox regression model was adjusted based on the variables that were considered clinically relevant or with p-values less than 0.05 in univariate analysis. ECW and TBW were excluded due to their direct correlation with EI. The multivariate Cox proportional model was analyzed with a forward stepwise method, with entry criteria set at p < 0.05. The adjusted hazard ratio (HR) and their 95% confidence interval (CI) were calculated. Statistical significance was defined as a two-sided p-value < 0.05. HR > 1.0 with a p-value < 0.05 indicated a deleterious association, while HR < 1.0 with a p-value < 0.05 indicated a protective association. Data were analyzed with SPSS version 25.0 (IBM, USA), MedCalc statistical software 19.2.6, and GraphPad Prism 8.4.3.

## 3. Results

From August 2019 to October 2021, a total of 409 consecutive patients were diagnosed with CHF, among which eight patients had incomplete data or loss of follow-up, and the remaining 401 patients with CHF were included in this study.

**Figure 1:**
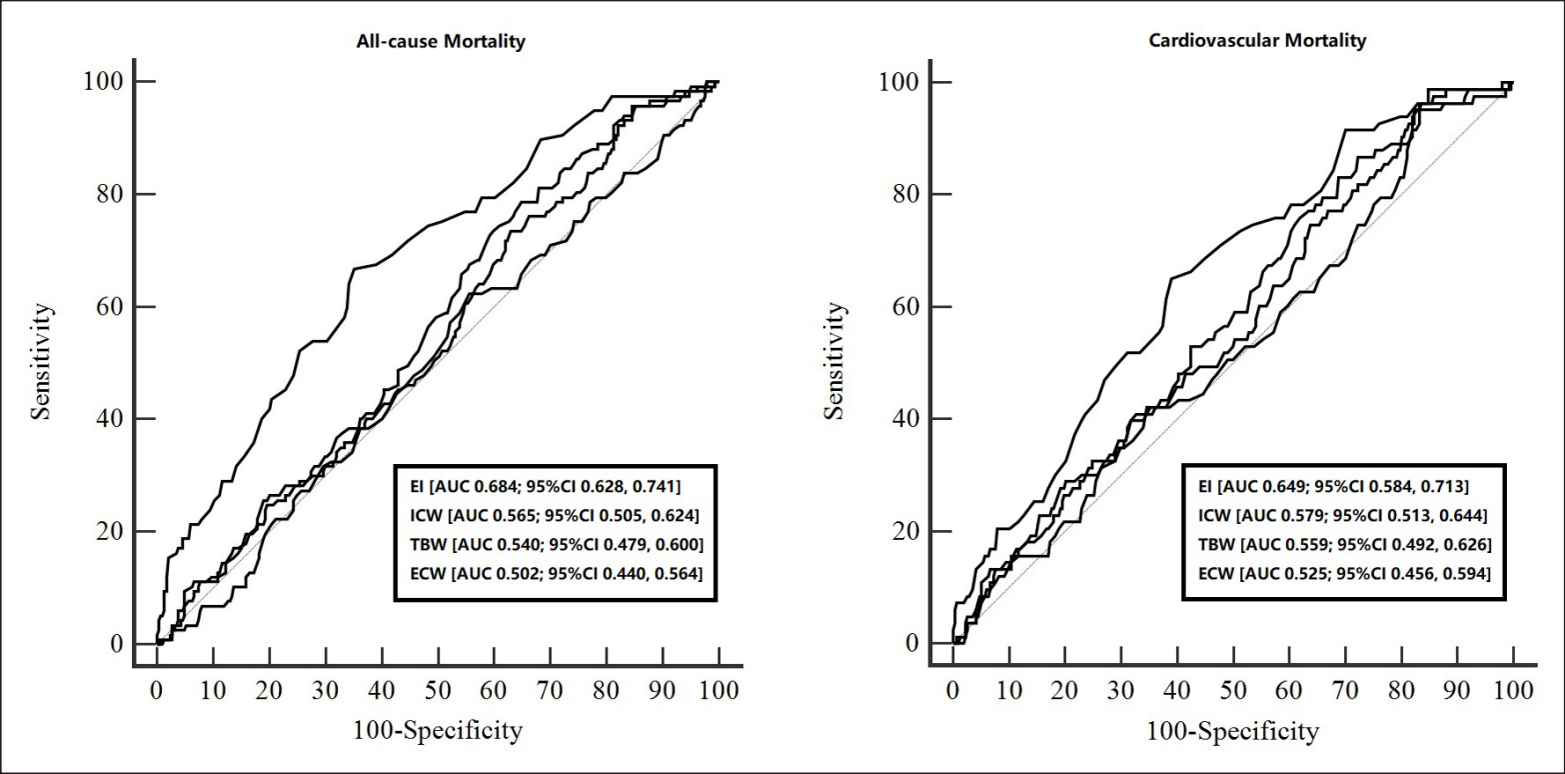
ROC analysis of edema index (EI), intracellular water (ICW), extracellular water (ECW), and total body water (TBW) for predicting long-term all-cause and cardiovascular mortality.

In Figure 1, we assess the sensitivity and specificity of ECW, TBW, and EI to predict long-term all-cause and cardiovascular mortality in patients with CHF. Based on our data, we found that EI (AUC 0.684; 95% CI 0.628, 0.741 and AUC 0.649; 9% CI 0.584, 0.713, respectively) has the highest predictive value compared to ICW (AUC 0.565; 95% CI 0.505, 0.624 and AUC 0.579; 95% CI 0.513, 0.644, respectively), TBW (AUC 0.540; 95% CI 0.479, 0.600 and AUC 0.559; 95% CI 0.492, 0.626, respectively), and ECW (AUC 0.502; 95% CI 0.440, 0.564 and AUC 0.525; 95% CI 0.456, 0.594, respectively). The Youden index was calculated based on the present study data and found that the optimal cut-off value for EI was 0.397, with a sensitivity of 66.67%, and a specificity of 64.79%. Furthermore, we divide the study population into two groups: low EI (≤0.397) and high EI (>0.397) groups. The baseline characteristics between the two groups were analyzed as follows.

Table 1 shows the baseline demographic characteristics between the two groups. The patients in the high EI group tended to be older and presented with atrial fibrillation (all p < 0.05). As for medical histories, a greater proportion of patients in the high EI group presented with a history of diabetes mellitus and chronic kidney disease (all p < 0.05), but there was no difference for hypertension, previous percutaneous coronary intervention (PCI), previous coronary artery bypass grafting (CABG), and chronic obstructive pulmonary disease (COPD) (all p > 0.05). Moreover, in terms of laboratory parameters and echocardiography, the high EI group was more likely to have lower hemoglobin and platelet, higher N-terminal pro-B type natriuretic peptide (NT-proBNP), higher D-dimer, creatinine, and a larger right atrium, right ventricle, and left atrium (all p < 0.05). Moreover, a higher percentage of patients presented with moderate to severe mitral regurgitation in the high EI group (p = 0.010).

**Table 1:** A comparison of baseline characteristics of the 2 groups.


BASELINE CHARACTERISTICS	TOTAL	EI ≤ 0.397	EI > 0.397	p VALUE

	n = 401	n = 223	n = 178	

Age (years)	69.51 ± 13.74	66.02 ± 14.26	73.88 ± 11.70	<0.001

Male (%)	249 (62.1)	140 (62.8)	109 (61.2)	0.708

BMI (kg/m2)	23.97 ± 4.09	24.12 ± 3.69	23.76 ± 4.54	0.381

Smoking (%)	174 (43.4)	97 (43.5)	77 (43.3)	0.962

Alcohol drinking (%)	134 (33.4)	79 (35.4)	55 (30.9)	0.340

Family History of CVD (%)	52 (13.0)	29 (13.0)	23 (12.9)	0.980

Atrial Fibrillation (%)	160 (39.9)	71 (31.9)	89 (50.0)	<0.001

NYHA Class (%)				<0.001

1	29 (7.2)	26 (11.7)	3 (1.7)	

2	94 (23.4)	64 (28.7)	30 (16.9)	

3	185 (46.1)	96 (43.0)	89 (50.0)	

4	93 (23.2)	37 (16.6)	56 (51.5)	

Ischemic Heart Disease (%)	171 (42.7)	100 (44.8)	71 (39.9)	0.319

Dilated Cardiomyopathy (%)	64 (16.0)	43 (19.3)	21 (11.8)	0.042

Medical histories (%)				

Hypertension	206 (51.4)	109 (48.9)	97 (54.5)	0.264

Diabetes mellitus	131 (32.7)	61 (27.3)	70 (39.4)	0.040

COPD	29 (7.2)	14 (6.3)	15 (8.4)	0.409

Dyslipidemia	39 (9.7)	32 (14.3)	7 (3.9)	<0.001

Previous MI	46 (11.5)	27 (12.1)	19 (10.7)	0.654

Previous PCI	62 (15.5)	31 (13.9)	31 (17.4)	0.333

Previous CABG	3 (0.7)	2 (0.9)	1 (0.6)	0.699

Chronic Kidney Disease	104 (25.9)	43 (19.3)	61 (34.3)	<0.001

Laboratory Parameters				

Cardiac Troponin I (ng/mL)	0.05 (0.01–0.79)	0.05 (0.02–1.10)	0.05 (0.01–0.47)	0.064

NT-proBNP (pg/mL)	1500 (599–4045)	1.120 (450–3110)	2035 (1003–4700)	0.006

D-dimer	685 (233–1316)	520 (206–1150)	864 (430–1635)	0.001

Leukocyte (×10^9^/L)	6.56 (5.40–8.76)	6.78 (5.60–8.95)	6.36 (5.06–8.48)	0.057

Hemoglobin (g/L)	130 (113–145)	135.0 (123.0–147.5)	121.5 (104.0–141.3)	<0.001

Platelet (×10^9^/L)	172 (141–222)	184.0 (151.5–236.5)	164.5 (124.8–205.3)	<0.001

Urea (mmol/L)	8.1 (6.2–12.0)	7.1 (5.9–10.0)	9.8 (6.8–13.5)	<0.001

Creatinine (μmol/L)	93 (73–131)	87.0 (72.0–112.5)	105.5 (73.5–161.3)	0.003

Uric Acid (μmol/L)	427.5 (335.5–561.5)	410.5 (329.0–522.0)	453.0 (348.3–595.0)	0.003

Echocardiography				

Right Atrium (mm)	40.86 ± 6.98	39.43 ± 6.39	42.65 ± 7.29	<0.001

Right Ventricle (mm)	22.43 ± 4.12	21.87 ± 3.50	23.14 ± 4.70	0.002

Left Atrium (mm)	41.06 ± 8.42	39.96 ± 8.05	42.43 ± 8.69	0.003

LVEDD (mm)	57.34 ± 10.27	57.61 ± 10.07	57.01 ± 10.53	0.565

IVS (mm)	10.58 ± 2.78	10.53 ± 2.47	10.65 ± 3.14	0.688

Moderate to severe MR	183 (45.6)	89 (39.9)	94 (52.8)	0.010

FS (%)	24.16 ± 8.31	23.21 ± 7.54	25.41 ± 9.10	0.022

EF (%)	43.70 ± 12.87	42.74 ± 12.21	44.90 ± 13.59	0.096


* EI: edema index; BMI: body mass index; CVD: cardiovascular diseases; NYHA: New York Heart Association Functional Classification; COPD: chronic obstructive pulmonary disease; MI: myocardial infarction; PCI: percutaneous coronary intervention; CABG: coronary artery bypass graft surgery; NT-proBNP: N-terminal prohormone of brain natriuretic peptide; LVEDD: left ventricular end-diastolic diameter; IVS: intraventricular septum; MR: mitral regurgitation; FS: fractional shortening; EF: ejection fraction.

Segmental water distribution analyzed by BIA is presented in Table 2. TBW and ICW were comparable between the two groups, while ECW, waist circumference, protein, and total body water to lean body mass (TBW/LBM) ratio were statistically significant (all p < 0.05). Furthermore, the lower extremities’ EI was higher than that of the trunk and upper extremities (p < 0.001).

**Table 2:** A comparison of bioelectrical impedance analysis of the 2 groups.


	TOTAL	EI ≤ 0.397	EI > 0.397	p VALUE

	n = 401	n = 223	n = 178	

Height (cm)	160.96 ± 8.34	160.90 ± 8.14	161.03 ± 8.61	0.873

Weight (kg)	62.22 ± 13.05	62.95 ± 12.28	61.30 ± 13.93	0.210

Waist Circumference (cm)	77.12 ± 12.89	79.94 ± 12.57	73.60 ± 12.43	<0.001

Intracellular Water	20.55 ± 4.86	21.02 ± 5.04	19.96 ± 4.56	0.509

Extracellular Water	13.45 ± 3.13	12.97 ± 2.89	14.05 ± 3.31	0.043

Total Body Water	34.00 ± 7.81	33.99 ± 7.80	34.00 ± 7.83	0.692

Edema Index of upper extremities	0.384 ± 0.012	0.378 ± 0.012	0.391 ± 0.008	<0.001

Edema Index of trunk	0.396 ± 0.027	0.384 ± 0.015	0.415 ± 0.014	<0.001

Edema Index of lower extremities	0.398 ± 0.025	0.383 ± 0.022	0.417 ± 0.014	<0.001

Protein	8.89 ± 2.10	9.09 ± 2.18	8.62 ± 1.98	0.027

Mineral	2.83 ± 0.70	2.80 ± 0.74	2.87 ± 0.64	0.362

Muscle Mass (kg)	43.44 ± 10.01	43.64 ± 10.09	43.18 ± 9.93	0.652

Lean Body Mass (kg)	46.27 ± 10.61	46.44 ± 10.72	46.05 ± 10.50	0.713

TBW/LBM (%)	73.48	73.22	73.91	<0.001

Body Fat Mass (kg)	15.95 ± 8.48	16.51 ± 7.90	15.26 ± 9.14	0.142

Body Fat Percentage (%)	25.06	25.92	23.99	0.089


*EI: edema index; TBW: total body weight; LBM: lean body mass; TBW/LBM: total body weight to lean body mass ratio.

**Figure 2:**
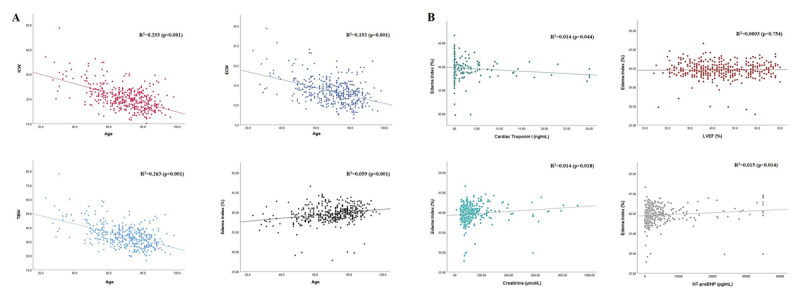
Correlation analysis. Figure 2A. Correlation analysis between age with intracellular water (ICW), extracellular water (ECW), total body water (TBW), and edema index (EI). Figure 2B Correlation between edema index (EI) with cardiac troponin I, left ventricular ejection fraction (LVEF), creatinine, and NT-proBNP.

Correlations between age and body fluid content are presented in Figure 2. A correlation analysis in Figure 2A shows that TBW (R^2^ = 0.263, p < 0.001), ECW (R^2^ = 0.193, p < 0.001), and ICW (R^2^ = 0.293, p < 0.001) content decreased with age, although the trend was predominantly observed in the ICW content. Moreover, the percentage of EI had a positive correlation with age (R^2^ = 0.059, p < 0.001). Figure 2B displays the correlation between EI and other CHF indicators, such as cardiac troponin I, left ventricular ejection fraction (LVEF), NT-proBNP, and creatinine. It revealed that EI had no significant correlation with LVEF (p = 0.754) but was negatively correlated with cardiac troponin (R^2^ = 0.014, p = 0.044). Moreover, EI was positively correlated with creatinine (R^2^ = 0.014, p = 0.018) and NT-proBNP (R^2^ = 0.015, p = 0.014).

**Figure 3:**
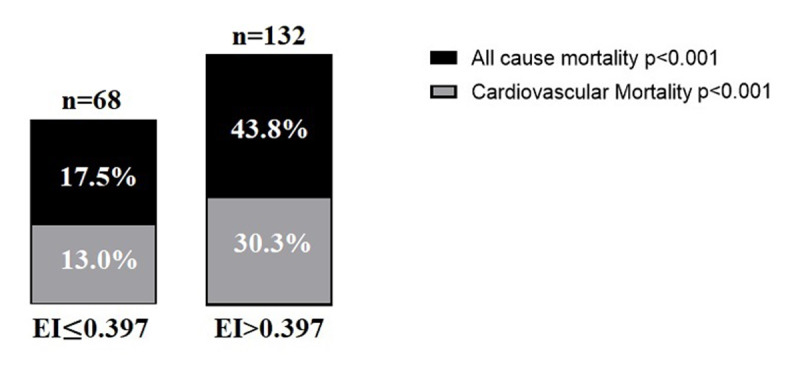
Long-term all-cause and cardiovascular mortality according to the edema index (EI).

**Figure 4:**
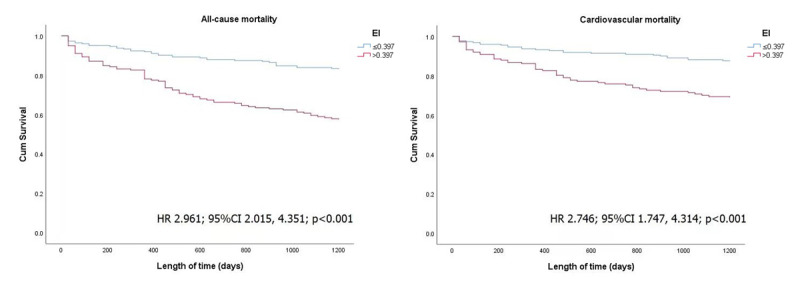
Kaplan-Meier curves for all-cause and cardiovascular mortality in patients with CHF.

During a median follow-up of 1200 days, all-causes mortality occurred in 117 (29.2%) patients, among which cardiovascular mortality accounted for 83 (70.94%). Furthermore, in Figure 3, the all-cause and cardiovascular mortality rate was significantly higher in the high EI group compared to the low EI group (all-cause mortality rate 43.8% vs. 30.3%, p < 0.001, and cardiovascular mortality rate 17.5% vs. 13.0%, p < 0.001, respectively). Figure 4 shows the KM curves based on the cut-off value of EI. The cumulative all-cause and cardiovascular mortality was significantly higher in the high EI group (HR 2.961; 95% CI 2.015, 4.351; p < 0.001 and HR 2.746; 95% CI 1.747, 4.314; p < 0.001, respectively).

Cox regressions were performed to determine the association between EI and primary outcomes (Table 3). In the univariate Cox proportional hazard analysis, age, body mass index (BMI), New York Heart Association (NYHA) functional classification ≥ 3, ICW, TBW/LBM, and EI > 0.397 were associated with all-cause mortality (all p < 0.05). While in terms of cardiovascular mortality, the covariates were almost the same, except for TBW/LBM. Furthermore, in the multivariate Cox proportional hazard analysis, EI > 0.397 was an independent predictor for both all-cause mortality (HR 1.959; 95% CI 1.304, 2.944; p = 0.001) and cardiovascular mortality (HR 2.051; 95% CI 1.276, 3.296; p = 0.003). The other independent factors for all-cause mortality were age, male, BMI, and NYHA ≥ 3, while for cardiovascular mortality, independent factors were age, BMI, NYHA ≥ 3, and LVEF ≤ 50%.

**Table 3:** Univariate and multivariate cox regression analysis of all-cause mortality **(A)** and cardiovascular mortality **(B)**.


	UNIVARIABLE		MULTIVARIABLE	
	
	HR (95% CI)	p	HR (95% CI)	p

(A) All-cause Mortality				

Age (1 year increase)	1.045 (1.028, 1.062)	<0.001	1.036 (1.018, 1.054)	<0.001

Male	1.367 (0.926, 2.018)	0.116	1.633 (1.092, 2.441)	0.017

BMI	0.920 (0.876, 0.968)	0.001	0.947 (0.903, 0.994)	0.029

NYHA ≥ 3	2.773 (1.678, 4.583)	<0.001	2.148 (1.290, 3.578)	0.003

Atrial Fibrillation	1.092 (0.755, 1.578)	0.640		

NT-proBNP ≥ 450 pg/ml	1.621 (0.943, 2.788)	0.081		

LVEF ≤ 50%	1.068 (0.711, 1.603)	0.752		

Moderate-Severe MR	1.386 (0.964, 1.992)	0.078		

Intracellular Water	0.952 (0.914, 0.992)	0.018		

TBW/LBM	1.452 (1.113, 1.895)	0.006		

Edema Index > 0.397	2.961 (2.015, 4.351)	<0.001	1.959 (1.304, 2.944)	0.001

(B) Cardiovascular Mortality				

Age (1 year increase)	1.031 (1.012, 1.050)	0.001	1.026 (1.005, 1.047)	0.016

Male	1.550 (0.964, 2.491)	0.070		

BMI	0.900 (0.848, 0.956)	0.001	0.919 (0.866, 0.974)	0.005

NYHA ≥ 3	2.975 (1.613, 5.486)	<0.001	2.277 (1.223, 4.239)	0.009

Atrial Fibrillation	1.090 (0.704, 1.688)	0.700		

NT-proBNP ≥ 450 pg/ml	1.555 (0.824, 2.933)	0.173		

LVEF ≤ 50%	1.683 (0.976, 2.904)	0.061	2.203 (1.251, 3.882)	0.006

Moderate-Severe MR	2.059 (1.320, 3.212)	0.001		

Intracellular Water	0.938 (0.892, 0.986)	0.012		

TBW/LBM	1.291 (0.941, 1.771)	0.113		

Edema Index > 0.397	2.746 (1.747, 4.314)	<0.001	2.051 (1.276, 3.296)	0.003


*NYHA: New York Heart Association Functional Classification; NT-proBNP: N-terminal prohormone brain natriuretic peptide; LVEF: left ventricular ejection fraction; MR: mitral regurgitation; TBW/LBM: total body weight to lean body mass ratio.

**Figure 5:**
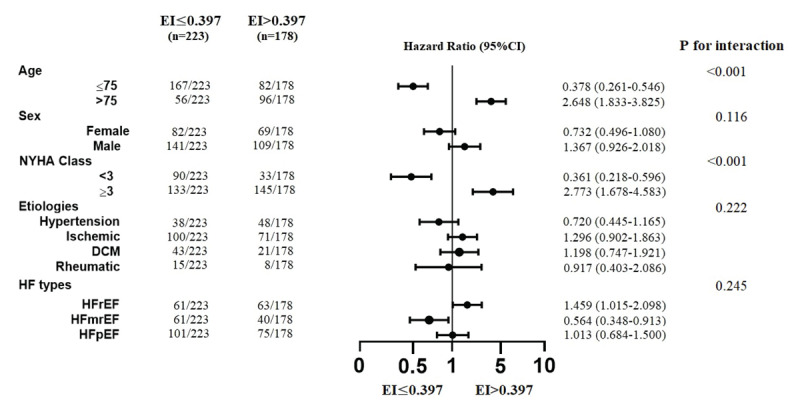
Forest plot of primary outcome according to the demographic characteristics.

For a more detailed analysis of the association between EI and all-cause mortality in CHF, subgroups were analyzed (Figure 5). Results indicated that EI > 0.397 was associated with an increased risk of long-term mortality, especially in patients with age >75 years and NYHA class ≥ 3 (all p < 0.05).

## 4. Discussion

The main findings from our present study are as follows. First, BIA can be used as a simple, reliable, and non-invasive tool to evaluate the congestion status of patients with CHF. Among the parameters obtained from BIA, EI had a relatively higher predictive value for long-term outcomes. Second, high admission EI was associated with an increased risk of long-term all-cause mortality and cardiovascular mortality. Third, after multivariable adjustment, high EI was an independent risk factor for long-term all-cause mortality and cardiovascular mortality, especially in patients with age >75 years old and NYHA class ≥3.

Sodium and water retention is one of the cardinal features in patients with HF. A decrease in cardiac output is the primary cause of fluid retention. In early compensated stages of CHF, elevated left ventricular end-diastolic volume secondary to both decreased cardiac output and ECW expansion leads to increased stroke volume and the restoration of cardiac output. At this stage, EI in patients with CHF is higher than healthy control [12]. As CHF progresses, low effective blood volume causes progressive salt and water retention, which aggravates ECW expansion. Determining the optimal ECW level and maintaining the patient at the optimal level are major management goals. However, precisely evaluating the fluid status in patients with HF is usually very challenging. Traditionally, monitoring patients’ weight, physical signs, and cardiac biomarkers, such as plasma BNP level, are commonly used to assess the fluid status and plasma volume in patients with CHF [13]. Nevertheless, these methods only give a rough estimation of body fluid content and are often insensitive, which may provide inadequate warning of impending decompensation [14]. Although invasive hemodynamic monitoring, such as right heart catheterization, could provide relatively accurate parameters associated with fluid status, it cannot be widely used in patients with CHF.

During the past decades, new noninvasive techniques, such as dual-energy X-ray absorptiometry (DXA) [15], magnetic resonance imaging (MRI) [16], and BIA [17] were developed to measure body composition. Each method has its advantages and disadvantages. DXA is considered the gold standard for body composition assessment and is the most accurate way to measure body fat, lean muscle mass, and bone density [18]. However, it requires specialized equipment and is moderately expensive; therefore, it is now often used as a reference method to compare with other body composition assessment methods. MRI can provide whole-body composition information, especially for estimating fat and skeletal muscle [19]; however, it is time-consuming and expensive, limiting its wide use in clinical practice. In contrast, BIA is inexpensive, can be easily used for noninvasive indirect assessment of body composition, and offers a variety of outcome parameters, including ICW, ECW, and TBW [20], which are critical parameters for fluid distribution assessment. In addition, BIA’s high sensitivity, short testing time, and convenience for bedside use make this device a practical body composition assessment method, most notably in the clinical setting. Moreover, studies have shown that the accuracy of BIA for body composition assessment was non-inferior to that of DXA [21,22,23], indicating the validity of BIA in assessing body composition in daily practice.

Although BIA provides multiple information on body composition, including fluid distribution, body fat, muscle mass, protein, and inorganic salt [24], our present study mainly focused on the fluid parameters because fluid retention is a critical issue in the treatment of HF. In addition, precise evaluation of the volume status is challenging without invasive monitoring. Therefore, it is of great importance to find a simple and effective method or index that can precisely reflect the volume status in patients with HF. Based on the pathophysiological process involved in HF, in our present study, we evaluated the features of fluid distribution and the prognostic impact of fluid change with fluid parameters obtained from BIA testing in patients with CHF.

CHF is common in the elderly and is often associated with fluid overload. However, conclusions regarding fluid changes in patients with CHF remain inconsistent. In our present study, it was found that TBW, ICW, and ECW gradually decreased with age. In contrast, Tai et al. [25] found that ECW tended to increase with age. The possible explanation for this disparity is the different enrolled patients. Our present study enrolled patients with CHF, while in Tai et al.’s study, the involved patients were diagnosed with chronic kidney disease. Moreover, in our present study, patients with CHF usually need diuretics, which can cause reduced ECW. Thus, they tended to present with lower levels of ECW. Nonetheless, we found that EI increased with age, suggesting that EI is superior to ECW or TBW in terms of evaluating the fluid status of elderly patients with CHF.

Previous studies have shown BIA analysis provided important information for the therapy in patients with HF. De et al. [26] conducted a study on patients with acute HF and found that BIA was a promising approach to detect changes in hydration status in patients undergoing intensified diuretic therapy. Soderberg et al. [27] evaluated the feasibility of BIA in congestive HF patients undergoing diuretic treatment and found that weight loss during diuretic treatment was mainly due to loss of ECW, while ICW had no significant changes. Based on the aforementioned findings, EI, as the ratio of ECW to TBW, was supposed to be an important marker of evaluating the fluid retention status. In fact, this index has been extensively studied in other diseases, such as cancer [9, 28], peritoneal dialysis [10], and congenital heart disease [29]; however, evidence regarding the prognostic value of EI in patients with CHF remains scarce. Liu et al. [30] reported that an EI of more than 0.390 was a predictor of HF readmissions and all-cause mortality in patients with acute decompensated HF. A recent study by Namba et al. [31] found that high EI before discharge (>0.408) was significantly associated with worse outcomes, including all-cause mortality and readmissions in HF patients. To the best of our knowledge, this is the first study to evaluate the prognostic value of EI in patients with CHF. Consistent with previous findings, our study demonstrated that admission EI was independently associated with long-term outcomes in patients with CHF. Our present study confirmed and extended previous findings, suggesting EI was an important prognostic factor in patients with CHF. It is noteworthy that no fixed reference value for EI is used in clinical practice, ranging from 0.370 to 0.408, depending on the disease and participants involved [9, 10, 30, 31, 32]. The optimal cut-off value of EI in our present study was 0.397, higher than in other studies [9, 10, 30, 32]. The possible interpretation may be that the extent of change of ECW in patients with CHF was more significant than in other diseases. However, whether the cut-off value in our study can be generalized deserves further study with a large sample size.

Our present study has several clinical implications. First, fluid congestion is one of the important pathophysiologic mechanisms of HF; thus, assessment of fluid retention is of great importance to guide the decongestion strategy, and BIA can be used as a non-invasive, inexpensive, safe, and reliable method to assess the fluid status and composition for patients with CHF. Second, given that EI was independently associated with the long-term outcome, it could be regarded as a simple risk stratification tool and prognostic biomarker for patients with CHF. Third, taking previous and present findings together, EI-guided management may be a promising way to achieve and maintain euvolemia in patients with CHF.

## 5. Limitations

The present study has some limitations. First, this is a single-center prospective observational study with limitations such as selection bias and potential confounders. Second, although BIA was performed on all patients within eight hours of admission, there are diurnal variations in data obtained by BIA. Third, BIA technology has some limitations, especially in the following conditions: firstly, obese patients tend to have an excess of fat mass and a higher ratio of ECW to TBW, which affects the accuracy of BIA [33]; secondly, the body’s hydration level is important for the accuracy of BIA. Unfortunately, patients with CHF often present with volume overload, and inappropriate use of diuretics may cause dehydration, which causes fat-free mass, including the underestimation of muscle and bone [34]. In addition, studies have shown that body composition changes after food or drink consumption [35]. Thus, BIA testing is recommended after overnight fasting [36]. The potential limitations of BIA could affect the accuracy applied to our patients. Fourth, we only tested the admission index while fluid status changes rapidly with meals, excretion, exercise, and the use of diuretics, so a series of follow-up examinations may provide better information. Fifth, we are aware that measuring pulmonary capillary wedge pressure and right atrial pressure by cardiac catheterization is the golden standard to evaluate one’s volume status; however, this was a prospective observational study with the aim to evaluate the association between EI obtained from BIA and the long-term outcome in patients with CHF—and not all patients underwent right heart catheterization, thus lacking the golden standard control. Further studies are needed to clarify the relationship between EI with pulmonary capillary wedge pressure and right atrial pressure. Lastly, the cut-off value of EI was calculated based on the study sample size, and the sample size of our study was relatively small. Hence, careful interpretation and studies with larger sample sizes are still required to confirm our findings.

## 6. Conclusions

Admission EI could be used as a new biomarker for predicting long-term mortality in patients with CHF, and higher EI was associated with an increased risk of all-cause and cardiovascular mortality. Furthermore, EI-guided management could be a promising therapy in patients with CHF.

## Data Accessibility Statement

The data that supports the findings of this study are available from the corresponding author upon reasonable request.
